# The mediator role of treatment response on oral health related quality of life in Behçet’s syndrome

**DOI:** 10.4317/medoral.26319

**Published:** 2023-12-27

**Authors:** Elif Naz Altıngöz, Yıldız Yenisoy, Aysun Kapusuz, Kerem Abacar, Nur Şişman-Kitapçı, Meral Yay, Umit Karacaylı, Fatma Alibaz-Öner, Nevsun İnanç, Tulin Ergun, Farida Fortune, Haner Direskeneli, Gonca Mumcu

**Affiliations:** 1 MSc student, Institute of Health Sciences, Medical School, Istanbul, Turkey; 2 Department of Rheumatology, School of Medicine, Marmara University, Istanbul, Turkey; 3 Faculty of Health Sciences, Marmara University, Istanbul, Turkey; 4 Department of Statistics, Science and Letters Faculty, Mimar Sinan Fine Art Faculty, Istanbul, Turkey; 5 Department of Oral and Maxillofacial Surgery, Faculty of Dentistry, Health Sciences University, Ankara, Istanbul, Turkey; 6 Department of Dermatology, School of Medicine, Marmara University, Istanbul, Turkey; 7 Barts and The London School of Medicine and Dentistry, Centre Immunobiology and Regenerative Medicine, Queen Mary University of London, UK

## Abstract

**Background:**

The aim of the study was to analyse the effects of Treatment Response with oral ulcers on oral health related quality of life in Behçet’s syndrome (BS).

**Material and Methods:**

In the cross-sectional study, 339 BS patients (F/M: 179/160, mean age: 36,13±9,81 years) were included. Data were collected by clinical examinations and patient reported outcome measures (PROMs) regarding Oral Health Impact Profile-14 (OHIP-14) questionnaire and self-reported Treatment Responses coded by a 5-point Likert-type scale (1: symptoms were cured- 5: symptoms were worsened). Moderated Mediation analysis (MA) was used to understand how oral ulcer activity (independent variable; X) influenced OHIP-14 score (outcome variables, Y) through self-reported Treatment Response (M1) and age (M2) as possible mediator variables (M) and disease course (mucocutaneous and musculuskeletal involvement vs. major organ involvement) as a possible moderator variable (W) on these relationships.

**Results:**

In Moderated MA, OHIP-14 score (Y) was mediated by the presence of oral ulcer (X) (*p*=0.0000), the negative Treatment Response (M1) (*p*=0.0001) and being young (M2) (*p*=0.0053) with mucocutaneous involvement (W)(p=0.0039).

**Conclusions:**

Self-reported Treatment Response as an underestimated issue has a Mediator role in relation to oral ulceration on oral health related quality of life in the framework of patient empowerment strategies. Therefore, study results give clues to assist physicians and dentists for better understanding of patients’ perspective.

** Key words:**Treatment response, outcome measure, patient-centred care, patient empowerment, oral ulcer, Behçet’s syndrome.

## Introduction

Behçet’s Syndrome (BS) presents oro-genital ulcers, cutaneous and musculoskeletal manifestations and major organ involvement regarding neurological, ocular, vascular and gastrointestinal manifestations. Oral ulcers are main inhibiting factor for remission in most patients, especially in mucocutaneous involvement (1,2,3).

Currently, shifting from a traditional disease-focused to patient-centred approach is a fundamental issue for improving health outcomes in a chronic disease management (1,2,4,5). Disease-oriented outcomes focus on objective criteria in clinical practice from a physician's perspective. However, patient-reported outcome measures (PROMs) provide information about patients’ perspective in the clinical practice (4,6,7). Treatment Response also reflects whether treatment protocols meet patients’ needs or not (8,9). Using PROMs with relevant questions cover a wide range of disease manifestations or focus on some manifestations to detect modifications within patients and differences among patients in BS (1,2,10-12). Among them, Oral Health Impact Profile-14 (OHIP-14) as a generic oral health related quality of life (OHRQoL) questionnaire focuses on the effects of oral health problems (7) and addresses patients’ needs with oral ulcers in BS (12). Although PROMs are commonly used in clinical practice to clarify priorities for treatment planning, self-reported Treatment Responses are an underestimated issue in clinical trials and follow-up periods in the framework of patient-centred care, especially heterogenic clinical profile in BD. At this point, Mediation analysis (MA) as a statistical method helps to understand the causal sequences and underlying mechanisms in complex relations (13,14). Therefore, the aim of the study was to analyse the effects of Treatment Response with oral ulcer activity on OHRQoL by using mediation analyses in BS.

## Material and Methods

In this study, 339 patients with BS who had been diagnosed according to the ISG criteria (15) and followed in the Behcet’s Disease Outpatient Clinic of the Marmara University Medical School in Istanbul were included (Table 1). Data were collected by clinical examinations and a questionnaire between 2017 and 2019. The study was performed according to the principles of the Declaration of Helsinki and was approved by the Ethical Committee of Marmara University Institute of Health Sciences Ethics Committee [20.06.2019-152]. Informed consent was obtained from all patients. The inclusion criteria were being ≥18 years, under medical control for BS and accepting to participate the study while presence of other chronic conditions leading to BS like manifestations were the exclusion criteria. After calculating the disease severity score according to organ involvement (16), patients were categorised as having mucocutaneous and musculoskeletal involvement (53,4%) and major organ involvement (46,6%). Treatment protocols of patients were grouped as immunosuppressive/immunomodulator ones (ISs: azathioprine, corticosteroids, anti-TNF agents and interferons; 50,1%) and non-ISs: colchicine, salazopyrine, NSAIDs, antibiotics; 49,9%) (Table 1).

**Table 1:**
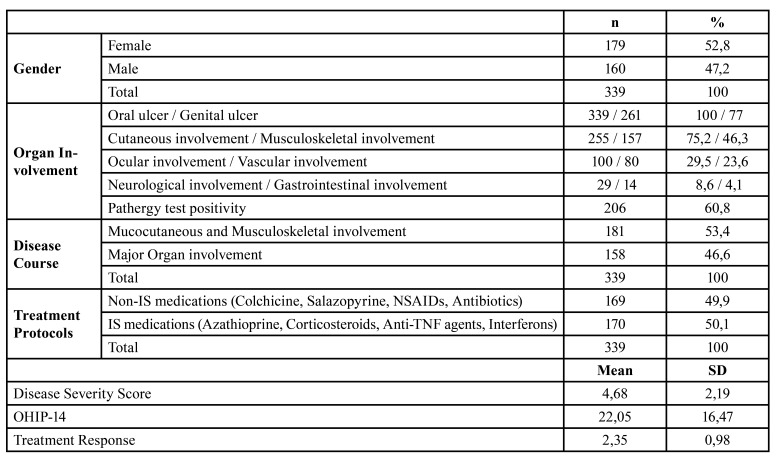
The profile of patients with Behçet’s syndrome.

The score of Oral Health Impact Profile-14 (OHIP-14) as a PROM is between “0 point (the best score)” and “56 points (the worst score)”in BS (12). Self-reported Treatment Responses were coded by a 5-point Likert-type scale (1: symptoms were completely cured vs 5: symptoms were worsened).

- Statistical Analysis

Analyses were performed by using SPSS 28.0 statistic program (SPSS Inc, Chicago, IL, USA). Mann-Whitney U test and Spearman correlation test were used due to the non-normal distribution of data. In addition, categorical variables were analysed by using the Chi-square test. In the study, p≤0.05 was accepted as statistically significant. Chronbach’s alpha value for internal reliability was 0,960 for OHIP-14.

Mediation analysis (MA) tests how an independent variable (X) influences the outcome variable (Y) through a third variable known as the mediator variable (M). Moderated MA also analyses the influence of a moderator variable (W) on these relationships (13,14). Independent variable (X) was oral ulcer activity (active: 1 vs. inactive: 0) in the study. Dependent variables (Y) was OHIP-14 score for oral ulcer activity. According to the preliminary analysis, Treatment Response (M1) coded by a 5-point Likert-type scale (1: symptoms were completely cured vs 5: symptoms were worsened) and age (M2) were thought to be possible mediators whereas disease severity (mucocutaneous and musculoskeletal involvement vs. major organ involvement) was defined as possible moderator variable (W). PROCESS macro was adopted in SPSS 28.0 for the MAs.

## Results

In this cross-sectional study, data from 339 patients with BS (F/M: 179/160; mean age: 36.13±9.81 years) were included. The disease severity score was found to be 4.68±2.19 in the group (Table 1). The mean number of visits during the last year and disease duration were 3.42±2.80 and 8,43±6,62 years. In addition, the mean duration from last dental visit was 24,63±4,0 months in the group.

Oral ulcer activity was seen 66,3% (*n*=225) in the group. The number and healing time of oral ulcers were 2,79±2,73 and 7,20±4,33 days in active patients. Patients with active oral ulcers were younger than inactive patients in the mucocutaneous and musculoskeletal involvement group (*p*=0.0011) whereas a similar relation was not seen in the major organ involvement group (*p*=0.233)(Table 2). Scores of OHIP-14 and self-reported Treatment Response were found to be high in patients with active oral ulcers compared to those of inactive patients in both disease course (*p*<0.05)(Table 2). The score of Treatment Response was found to be high in females (2,46±0,95 vs.2,22±1,01), patients with mucocutaneous and musculoskeletal involvement (2,49±0,91 vs. 2,17±1,04) and patients treated with non-IS treatment protocols (2,50±0,91 vs. 2,18±1,03) compared to those of others (*p*<0.05).

**Figure 1:**
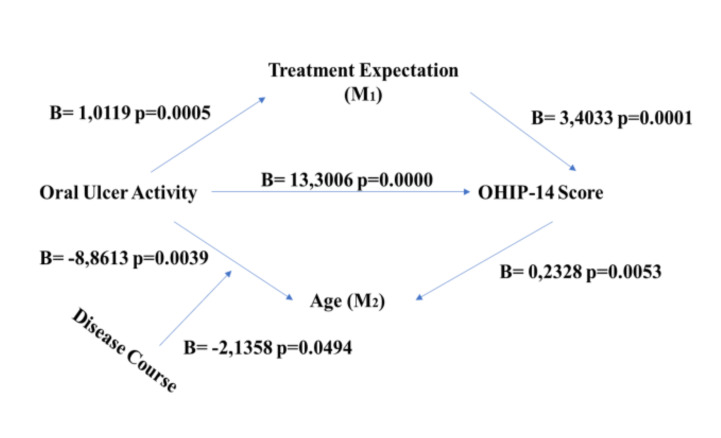
Moderated mediation analysis for OHIP-14 score in Behçet’s Syndrome.

In Moderated MA, OHIP-14 score (Y) was mediated by the presence of oral ulcer (X) directly (*p*=0.0000). The negative Treatment Response (M1)(p=0.0001) and being young patients (M2)(p=0.0053) in mucocutaneous involvement (W)(p=0.0494) were mediators in the model (Table 3)(Fig. 1). According to a bootstrap analysis with 5000 replications, the model was found to be significant in the study.

**Table 2:**
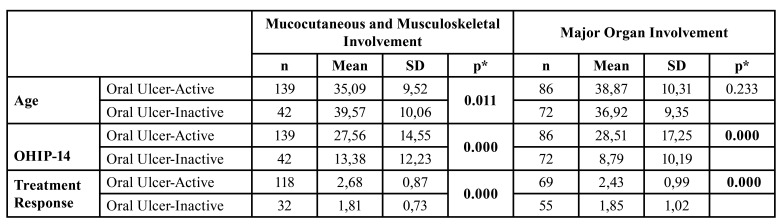
Oral ulcer activity and PROMs according to disease course.

**Table 3:**
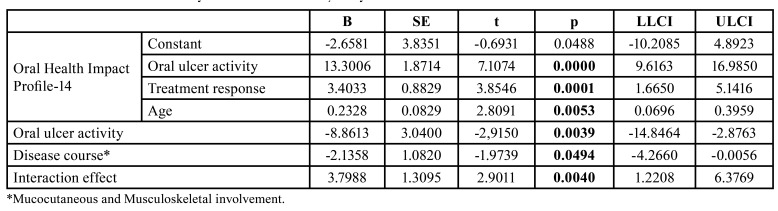
Moderated mediation analysis for OHIP-14 in Behçet’s syndrome.

## Discussion

The feedbacks and responses of patients are critical aspects for shared decision-making in patient-centred care (17). PROMs have benefits for clinicians to understand patients’ needs and the influential factors for the best clinical practice (6,10,11,18). In this study, the aim was to analyse the effects of Treatment Response on oral health related quality of life by using Mediation analyses in BS.

Most of the patients with BS had active oral ulcers and negative self-reported Treatment Response. Oral ulcer activity is commonly seen in mucocutaneous involvement and also thought to be risk factor for more severe disease course (2,19,20). Since oral ulcers lead to pain and functional limitation (21,22), patient’s life is poorly affected by them in BS (23). When remission couldn’t be achieved for oral ulcers, this condition leads to negative patient experiences easily. Therefore, the evaluation of self-reported treatment response is not underestimated for clinical trials.

In Moderated MA for OHIP-14, the elevated score was directly mediated by oral ulcer activity. Young patients with mucocutaneous and musculoskeletal involvement and negative Treatment Response were mediators for poor OHIP-14 score. These results were expected in BS patients as oral ulcers lead to poor oral health and quality of life and daily activity impairment (1,12,24-26). Moreover, disease manifestations may be more active in young patients with mucocutaneous involvement since aggressive treatments could not be used as firstline treatment protocols in this disease spectrum (3,19). Therefore, patients may have negative emotions during the disease progress because patients’ beliefs and emotional responses are poorly affected by the disease symptoms in the chronic disease management (27,28).

The score of Treatment Response was found to be poor in female patients who had mucocutaneous and musculoskeletal involvement treated with non-IS medications. Aggressive treatments are not used for female patients who don’t have mortality risk (2,3,19), except resistant cases in this spectrum (29). Since remission couldn’t be achieved by non-IS medications easily, negative Treatment Response could be predicted in this group. In addition, negative Treatment Response was also found to be a significant mediator for poor OHIP-14 score in the study. Although PROMs are commonly used in clinical practice to clarify priorities for treatment planning, negative Treatment Response is an underestimated issue in clinical practice. Nowadays, holistic approach to chronic disease management contains patient empowerment that increases self-management capability of patients to cope with their chronic diseases. Patients need health information related to treatment options and potential impact on their future health. When physicians recognise patients’ needs and experiences, they could help patients to cope with their health issues. At this point, positive Treatment Response as underestimated issue may be achieved by patient empowerment strategies that are enabled by the good communication between patients and physicians, the identification of patient’s responses, and providing information about patients’ needs and disease manifestations (30). Therefore, these results gave clues to assist physicians for better understanding of patients’ perspective, supporting patients with shared knowledge and accepting collaborative role of patients in the patient-centred care. Besides, level of health literacy of patients living with chronic disease is the essential point in shared decision-making for the patient-centred care. Therefore, it is necessary to close the gap between physician readiness and patient’s health literacy for the success of patient-centred care (17). Since the main limitation of the study was that data was collected at a single time point in a sole centre, further studies are necessary to assess Treatment Response with patient empowerment strategies at follow-up periods.

Consequently, since Treatment Response has a negative Mediator role in relation to oral ulceration on the PROMs, shared knowledge and shared decision-making could improve well-being of patients in the framework of patient-centred care.
